# Primary Care Considerations for Youth Martial Arts Athletes

**DOI:** 10.1007/s12178-024-09942-7

**Published:** 2025-01-09

**Authors:** Celina de Borja, Raechelle Ocampo, Ameera Teal

**Affiliations:** 1 Division of Pediatric Orthopaedics, University of California, 1825 4th Street– 5th Floor, San Francisco, CA 94158 USA; 2 Department of Pediatrics, University of California, San Francisco, USA

**Keywords:** Martial arts, Youth athletes, Injury prevention, Sports medicine

## Abstract

**Purpose of Review:**

This review aims to analyze the impact of martial arts on youth, addressing the physical and psychosocial benefits, as well as the injury risks associated with increased participation.

**Recent Findings:**

Data from the National Federation of State High School Associations indicates a notable increase in youth participation in martial arts, with a rise of over 13,000 participants from 2018 to 2022. In addition to physical benefits, recent studies highlight that martial arts can significantly enhance mindfulness, reduce stress, and improve self-control, thus diminishing bullying behaviors in school-aged children. Additionally, the therapeutic application of martial arts techniques has been effective in managing pain in children with chronic diseases and in alleviating emotional distress in their siblings. However, the popularity of the sport brings concerns over a spectrum of injuries, especially more severe cases in competitive settings.

**Summary:**

Martial arts provide a dynamic platform for fostering robust physical health and psychological resilience among youth. While the benefits are substantial, they are accompanied by risks that require careful management through informed practices and supervision. This review underscores the importance of understanding the dual nature of martial arts — its capacity to empower and its potential to harm — to maximize its benefits and safeguard young athletes.

## Introduction

Martial arts is a collective term for several styles of non-contact and contact combat sports. Non-contact martial arts emphasize form, technique, meditation, and self-control. Contact martial arts implement all non-contact components in addition to sparring where martial artists may execute grappling and/or striking techniques while training to prepare for competition [1]. The harmonious meditative elements and dynamic combat techniques of martial arts cater to a broad array of practitioners seeking either the serenity of a controlled environment or the thrill of competition [2]. While there are several martial arts disciplines, karate, taekwondo, kung fu, and judo are amongst the most practiced by youth martial artists [2, 3]. According to the National Federation of State High School Associations, the epidemiology of martial arts participation amongst youth and adolescents shows a significant uptick in participation, noting an increase of over 13,000 participants from 2018 to 2019 to 2021-2022 [4, 5]. However, this rise in popularity and participation is accompanied by a spectrum of injuries ranging from minor abrasions, lacerations, strains and sprains to more severe injuries such as fractures, joint dislocations, dental injuries, and concussions [2]. For parents and clinicians caring for youth martial arts athletes, it is imperative to have a thorough understanding of the sport’s demands as well as strategies for injury reduction to promote a safe and nurturing environment for youth martial artists seeking enjoyment in their practice.

## Benefits of Martial Arts

The benefits of martial arts for youth athletes are well documented [6–11]. A recent review by Stamenkovic et al. analyzed original research investigating the physical benefits of martial arts and found that martial arts improved cardiorespiratory fitness, strength, flexibility, and balance in martial artists aged 4 to 13 years old [10]. When compared to their non-athletic counterparts, youth athletes who participate in martial arts have shown increased aerobic capacity, muscular endurance, flexibility, and lower body fat percentage [12]. Martial arts are also effective in the clinical setting [6–8]. The implementation of Kicks Kicking Cancer, a martial arts therapy program administered by trained martial arts instructors was effective for pain management in children with chronic diseases, including malignancy [6]. Marusak et al. implemented the same martial arts therapy program and demonstrated similar improvements in pain, with the additional benefit of reduced emotional distress that extended to the siblings of children with chronic diseases and cancer [7]. These studies also highlight the psychosocial benefits of martial arts. Martial arts promote self-awareness using positive feedback, breathing techniques, and communal support [6–8, 13]. During the challenges brought forth by the pandemic, martial arts have contributed to increased mindfulness and decreased stress among students [6–8]. In addition, studies show children who participated in martial arts exhibit heightened self-control and lower tendencies towards bullying behaviors [9, 13]. 

## Injury Mechanisms

Understanding the different techniques used in specific martial arts practice is essential to knowing the issues and injuries associated with it [1]. Martial arts training involves non-contact (basic form and technique) and contact training. Non-contact martial arts disciplines such as Tai Chi and Qigong are a practice in controlled meditative movements that focus on the optimization of the mind and body connection to promote a positive impact on physical health and behavior [14]. In contrast, though non-contact, poomsae is combat-focused technical training within the taekwondo martial arts practice, where athletes demonstrate defense tactics without an opponent. The movements in poomsae can be effective in improving physical fitness for youth participants due to their high demand in explosive power, flexibility, and agility [15]. Martial arts disciplines that require contact utilize sparring. Sparring is used to practice movements in a controlled setting against an opponent. Age-based recommendations regarding when sparring should begin in martial arts vary and depend on the specific martial art, but in general, sparring is not typically recommended until children are at least 8–10 years old. In lieu of age-specific guidelines, the American Academy of Pediatrics, emphasize the importance of considering physical and cognitive development when determining if a youth is ready for sparring. Factors like the child’s maturity, ability to follow instructions, and physical preparedness for the intensity of sparring should be assessed before engaging in full-contact practice [2]. The purpose of sparring is to improve the timing and technique of movements in preparation for competition.

The movements, skills, and techniques required for successful engagement in contact training vary among martial arts disciplines. Likewise, the injury patterns associated with each martial arts discipline are closely related to the primary movements of the respective discipline. For example, karate, taekwondo, and kung fu are martial arts disciplines that primarily involve striking and blocking. Striking includes punching and kicking movements with the goal of self-defense [1–3, 16, 17]. These disciplines see an increased incidence of lower extremity injuries, particularly in the foot and ankle with the primary mechanism of injury being kicking, falling, and getting kicked [2, 3, 16–18]. Other martial arts disciplines, such as judo and mixed martial arts involve grappling techniques as a primary movement [1–3]. Grappling includes throws/takedowns choking and joint locking with the goal of forcing an opponent into submission [1–3]. Yard et al. found shoulder or upper arm injuries accounted for 19.1% of pediatric judo injuries presenting to the emergency department from 1990 − 2003 [3]. Most of these injuries were documented to be the result of being thrown/flipped while participating in judo. The specific nature of martial arts injury patterns may necessitate injury prevention strategies specific to the martial arts discipline. An extensive epidemiological study spanning from 2004 to 2021 elucidates the injury landscape further. Madireddy et al. observed higher injury rates in children aged 12 to 17 compared to those aged 3–11 with an elevated risk of head, neck, and traumatic brain injuries during competition amongst all ages [18]. The increased rate of injury in older martial artists may be attributed to their exposure time and experience level with more complex skills being demonstrated in higher level competitors [16, 17]. 

## Skin and Soft Tissue Injuries

The most common injuries in youth martial arts are skin and soft tissue injuries from direct trauma. The highest incidence of injuries includes abrasions and lacerations [3, 18]. Abrasions may be caused by friction or scraping against training mats, equipment, or opponents’ skin. Lacerations may result from blunt trauma, sharp objects, or contact with sharp edges of protective gear. Abrasions and lacerations should be cleaned and bandaged before returning to activity. The injury profile of youth martial artists may also include contusions and hematomas. Contusions may form from blunt force trauma, impact, or collision during sparring or striking techniques. Hematomas may form between skin or within soft tissues following trauma or vascular injury. Auricular hematoma, also known as cauliflower ear, is an injury seen commonly in martial arts [1]. Blunt trauma to the ear can cause blood to pool under the perichondrium. As a result, the cartilage is separated from the perichondrium, which causes the cartilage to form scar tissue and can become swollen. For this injury, it is important to drain the hematoma to resume flow of blood and nutrients to the tissue [19]. 

## Transmission of Infectious Diseases

Skin infection transmission is a significant concern in martial arts due to the physical nature of the sport, where participants frequently engage in close contact, such as grappling, ground fighting, and shared mat space. The most common skin infections in martial artists include bacterial infections like Staphylococcus aureus (including MRSA) and fungal infections like ringworm (Tinea corporis). Transmission occurs primarily through direct skin-to-skin contact or by contact with contaminated surfaces, such as mats, towels, or training equipment. MRSA infections are a concern in combat sports, as they can be easily transmitted in environments where athletes share equipment and sweat, creating an ideal medium for bacterial growth. Preventive measures, such as proper hygiene practices (e.g., showering immediately after training), routine cleaning and disinfection of mats and equipment, and the use of clean clothing, are essential in reducing the risk of transmission. In addition, monitoring and excluding athletes with visible skin infections from practice is a recommended strategy to prevent outbreaks [20]. 

## Acute Musculoskeletal Injuries

Sprains and strains are among the more common martial arts injuries [3, 18, 21]. Sprains are injuries that result from overstretching or tearing of ligaments while strains are the result of overstretching of musculature. These injuries are common in grappling techniques such as joint locks. For example, arm-lock techniques place the elbow in hyperextension and apply valgus stress which may result in anterior capsular or ulnar collateral ligament sprains [1]. Knee and ankle joint-locking, improper landing after jumping, and/or improper contact with an opponent may place the collateral ligaments at the knee and ankle at risk for sprains. In addition, punching can lead to jammed fingers and kicking that leads with the toes instead of the top of the foot or heel may lead to toe sprains. Management for strains and sprains includes rest from exercise and any physical activity that puts the injured area at risk of re-injury. Imaging the affected area may allow for a greater understanding of the extent of the injury and guide the patient care team in the appropriate treatment plan [22]. If the sprain or strain has not resolved with rest, the athlete may benefit from a rehabilitation program that outlines milestones for returning to activity administered by a physical therapist. While strains and sprains were the most common injury for martial artists aged 12–17 treated in emergency departments in the United States from 2004 to 2021, fractures and dislocations accounted for most injuries seen in martial artists aged 3–11 years old [18]. Dislocations should be reduced promptly to minimize the potential for sequelae and are best completed in the emergency department where pain control and/or sedation can be administered when appropriate. If a reduction is performed on site, the affected joint should be immobilized post-reduction, and the patient should receive radiographs to confirm proper alignment [23]. Careful consideration should be made for youth athletes with dislocations, as it is possible there could also be an accompanying fracture. The management strategy for pediatric fractures is multifactorial. Age, type of fracture (e.g. open vs. closed), site of injury, and severity of injury including displacement, involvement of growth plates or articular surface, are among the primary considerations in developing a treatment plan [24]. 

## Overuse Injuries

Repetitive microtrauma and cumulative stress on the musculoskeletal system can lead to overuse injuries [25–27]. Potential mechanisms for overuse injury in martial artists include repetitive punching, kicking, blocking, and grappling maneuvers that cause strain on the joints, muscles, tendons, and ligaments. Previous surveillance studies investigating the epidemiology of youth martial arts injuries are limited to competition and emergency department settings [3, 16, 18, 21, 28]. These studies do not account for injured athletes that presented to outpatient settings for treatment, nor do they include patients who failed to seek medical care altogether. Athletes experiencing overuse injuries oftentimes do not seek immediate medical attention. As a result, these injuries may be underestimated. Still, certain overuse injuries can put an athlete at risk for acute injury, such as stress fractures that can result in complete fracture. It is important for the healthcare team to be attentive to the health of the youth athlete as they may find significant enjoyment in their practice and resist time away from the sport in fear of being injured. Obtaining a thorough pre-participation evaluation is imperative to ensure the overall fitness of the youth athlete and their longevity in the sport [29, 30]. 

## Concussions

Although comprehensive data specific to concussions in youth martial arts are limited, emerging evidence suggests that concussions are not uncommon in this population [2]. Risk factors for concussions include age and developmental stage, training intensity and duration, technique and skill level, and equipment usage. Children and adolescents are more vulnerable to concussions due to ongoing brain development and immature neck musculature [18]. High-intensive training sessions, prolonged sparring, and frequent head impacts elevate the risk of concussions. The most common mechanism for concussions is direct strikes to the head from punches, kicks, elbows, or knees during sparring or competition. Other mechanisms include indirect impacts that cause sudden acceleration or deceleration of the head. Prompt recognition, evaluation, rest and recovery, and multidisciplinary care are important to minimize potential complications and facilitate safe return to activity [16, 17, 28]. 

## Guidance and Injury Prevention

Injury prevention in youth martial arts is essential and the following modifiable risk factors should be considered to ensure the continued participation and enjoyment of the sport.

### Equipment

Headgear is an essential piece of equipment that significantly decreases the likelihood of head and face injuries, such as abrasions, lacerations, and contusions. However, it is important to note that while headgear is protective from significant structural injuries, it does not lessen the risk of concussions. Therefore, proper technique and adherence to safety rules remain crucial in concussion prevention. Mouthguards also play a critical role in reducing dental and orofacial injuries. Body padding, especially in taekwondo, tends to benefit the athlete executing kicks, absorbing some of the impacts, and potentially reducing injury risk. This underscores the importance of appropriate protective gear tailored to the specific demands of each martial arts discipline [2]. 

### Strength and Conditioning

Resistance training for long-term youth athletic development is highly recommended for all active youth [31]. Research shows strength and conditioning programs designed and implemented by certified professionals can complement sport-specific training and reduce the likelihood of injury in youth athletes during practice and competition [32–34]. Recent research suggests that incorporating neck strength training into martial arts routines may help decrease the incidence of neck injuries. Strengthening the neck muscles could provide better stability and reduce the vulnerability of athletes to strains and other neck-related injuries [32]. Incorporating diversity of movement within training programs is essential to avoid overuse injuries, especially with activities that require repetitious movement like kicking in martial arts [35]. Kabadayı et al. added an eight-week bodyweight core strength program to the martial arts training of adolescent karate athletes and found it was effective in improving core endurance and karate kick performance [33]. Diverse movement is not limited to strength training. Yoo et al. found the addition of a proprioceptive training program improved balance in taekwondo poomsae athletes and resulted in improved athletic performance [36]. 

### Policies

Rule changes have shown mixed results; while limiting sparring in less experienced athletes has been recommended, sparring injuries are more commonly reported in experienced athletes due to stronger forces and higher technical abilities. In karate, for instance, directing strikes to allowed areas has reduced overall injuries in younger athletes, including head injuries, though it may have led to an uptick in leg injuries [2]. Injury risk can be minimized, however, when safety rules are strictly enforced in competition settings. Burke et al. found a low rate of injury during taekwondo competition (0.4/1000 athlete exposures) after on-site sports medicine physicians strongly impressed athlete adherence to safety rules on tournament judges [28]. 

### Technique and Supervision

Proper training of technique is fundamental for injury prevention. Emphasizing blocking skills in taekwondo, safety education, and strict enforcement of competition rules can significantly reduce the risk of concussion. Furthermore, it’s essential for instructors to provide proper supervision and ensure that athletes are engaging in safe practices [2]. Non-contact martial arts practices carry a lower risk for injury. Athletes should only progress to contact or competitive levels once they have demonstrated both physical and emotional maturity during non-contact training, along with competency in martial arts-specific movements and techniques. This phased approach allows athletes to develop their skills progressively and safely [2]. 

In summary, injury prevention in martial arts hinges on a combination of protective equipment, strength training, rule enforcement, technique education, and phased skill development. These components should form the foundation of any program designed to safeguard our young athletes as they pursue excellence in martial arts.

## Conclusion

The combative and meditative aspects of martial arts offer significant benefits to youth participants. Martial arts training may improve attributes of physical fitness including strength, balance, and flexibility in addition to enhancing cognitive function and emotional resilience through increased mindfulness, self-control, and stress reduction. Still, the prevalence of martial arts related injuries underscores the need for parents and clinicians to remain vigilant about safety practices, especially as participants’ age and competition intensifies. With appropriate supervision and adherence to safety guidelines, martial arts can serve as a powerful tool for personal development, fostering physical and psychological well-being in children and adolescents. By embracing both the challenges and opportunities that martial arts present, communities can help nurture a generation of disciplined, mindful, and healthy individuals.

## Data Availability

No datasets were generated or analysed during the current study.
